# Supplementary material for: miRBaseConverter: an R/Bioconductor package for converting and retrieving miRNA name, accession, sequence and family information in different versions of miRBase
Source: BMC Bioinformatics. 2018 Dec 31;19(Suppl 19):514. doi: 10.1186/s12859-018-2531-5 (PMC6311916; doi:10.1186/s12859-018-2531-5)
Supplement: Supplementary file 1 — The vignette of miRBaseConverter. (PDF 150 kb) [file 12859_2018_2531_MOESM1_ESM.pdf]

Additional file 1 — The vignette of miRBaseConverter

Contents

1 Overview . . . 2

2 miRNA Version check . . . 2

3 The conversion between miRBase Accession and miRNA Name . . . 3

3.1 miRBase Accession to miRNA Name . . . 3

3.2 miRNA Name to miRBase Accession . . . 4

4 The conversion of miRNA Names between two different miRBase versions . . . 4

4.1 Solution 1: Global searching and matching . . . 4

4.2 Solution 2: miRNA Names conversion with three steps . . . 5

5 The conversion between Precursor and Mature miRNA . . . 6

5.1 Mature miRNA to Precursor . . . 6

5.2 Precursor to Mature miRNA . . . 6

6 Retrieve the Family category of miRNAs . . . 7

7 Retrieve some of detailed miRNA information in miRBase . . . 8

7.1 Retrieve the Sequence of miRNAs . . . 8

7.2 Retrieve all the miRBase version information . . . 8

7.3 Retrieve all the available species in miRBase . . . 9

7.4 Retrieve all the available miRNAs in the specified miRBase version . . . 10

7.5 Retrieve all the history information of a single miRNA . . . 10

7.6 Retrieve the data table for the specified miRBase version . . . 14

8 The online retrieving of miRNA information . . . 15

8.1 Open the miRNA webpages in miRBase . . . 15

8.2 Open the miRNA family webpages in miRBase . . . 15

9 Conclusion . . . 16

1 Overview

miRBaseConverter is an R/Bioconductor package for converting and retrieving the definition of miRNAs (Names, Accessions, Sequences, Families and others) in different miRBase versions (From miRBase version 6 to version 22 [The latest version]). A tiny built-in database is embedded in the *miRBaseConverter* R package for retrieving miRNA information efficiently.

microRNAs(miRNAs) are one of the essential molecules that play the important role in the post-transcriptional gene regulation. The studies about novel miRNA and their function discoveries have an explosive growth in the last decade. The miRBase database is the authority archive of miRNA annotations and sequences for all species. With the development of researches about miRNAs, the annotation of miRNA has been changed significantly and develops many different historical versions. Each of the previous versions has been adopted in many research literatures and databases. Due to the inconsistent of name annotation of miRNAs, there is a barrier for the later scholars to reuse the previous research results in a convenient way, especially for miRNA databases with thousands of entries. There are some webservers or R-based tools can handle the batch conversion of miRNA names. However, an easy-to-use and well-documented tool for miRNA conversion and information retrieval is still lack. We present the *miRBaseConverter* R package, a comprehensive tool for miRNA research, to provide a suite of tools for checking miRNA Name, Accession, Sequence, version and family and history information. The *miRBaseConverter* package can be competent for all species including Precursor and Mature miRNAs defined in miRBase.

In the following sections, we present the detail usage of the functions included in the *miRBaseConverter* package.

2 miRNA Version check

For a list of miRNA names without version information, users may need to check what is the most possible miRBase version. *miRBaseConverter* package provides an easy-to-use function `checkMiRNAVersion()` to address this issue with an straightforward result.

```
library(miRBaseConverter)
data(miRNATest)
miRNANames = miRNATest$miRNA_Name
version=checkMiRNAVersion(miRNANames, verbose = TRUE)
##    Version Proportion           Recommend
## 1       v6     12.33%
## 2     v7_1     18.67%
## 3       v8     18.83%
## 4     v8_1     20.33%
## 5     v8_2      20.5%
## 6       v9        22%
## 7     v9_1        26%
## 8     v9_2        26%
## 9      v10        29%
## 10   v10_1      29.5%
```

```
## 11     v11      32.5%
## 12     v12        34%
## 13     v13     35.33%
## 14     v14      45.5%
## 15     v15     51.67%
## 16     v16     51.67%
## 17     v17     51.83%
## 18     v18     98.67%  ***BEST Matched***
## 19     v19     90.83%
## 20     v20     82.83%
## 21     v21      78.5%
## 22     v22     77.67%
```

The output text in console shows the matched proportions in all the miRBase version and gives the recommended version which is the best matched with the highest proportion values. This function is of great helpful miRNA version checking of a chunk of miRNAs.

3 The conversion between miRBase Accession and miRNA Name

3.1 miRBase Accession to miRNA Name

An Accession is the identifier that define miRNA uniquely in miRBase. Users can apply Accessions to retrieve the entire information of the miRNAs in miRBase. One of the most commonly used functions is to retrieve the corresponding miRNA name from Accession. The manual retrieval one by one in miRBase could be a tough work for a chunk of Accessions of interest. The function `miRNA_AccessionToName()` in *miRBaseConverter* package can conduct a high throughput transformation within quite short time.

```
library(miRBaseConverter)
data(miRNATest)
Accessions = miRNATest$Accession

#### 1. Convert to the Accessions to miRNA names in miRBase version 13
result1 = miRNA_AccessionToName(Accessions,targetVersion = "v13")
result1[c(341:345),]
##        Accession   TargetName
## 341 MIMAT0002843 hsa-miR-520b
## 342 MIMAT0001650  mtr-miR399c
## 343 MIMAT0000013   cel-miR-42
## 344 MIMAT0002885  osa-miR529a
## 345 MIMAT0011111         <NA>

####2. Convert to the Accessions to miRNA names in miRBase version 22.
result2 = miRNA_AccessionToName(Accessions,targetVersion = "v22")
result2[c(341:345),]
##        Accession      TargetName
## 341 MIMAT0002843 hsa-miR-520b-3p
```

```
## 342 MIMAT0001650     mtr-miR399c
## 343 MIMAT0000013   cel-miR-42-3p
## 344 MIMAT0002885     osa-miR529a
## 345 MIMAT0011111     mtr-miR169b
```

3.2 miRNA Name to miRBase Accession

The conversion of miRNA Name to Accession is the reversion process. Due to the frequent changes of miRNA name in different versions, researches are likely to adopt the miRBase Accessions as the identifiers in most literatures and databases.

```
library(miRBaseConverter)
data(miRNATest)
miRNANames = miRNATest$miRNA_Name
result1 = miRNA_NameToAccession(miRNANames,version = "v18")
result1[c(341:345),]
##     miRNAName_v18    Accession
## 341  hsa-miR-520b MIMAT0002843
## 342   mtr-miR399c MIMAT0001650
## 343 cel-miR-42-3p MIMAT0000013
## 344   osa-miR529a MIMAT0002885
## 345   mtr-miR169j MIMAT0011111
```

4 The conversion of miRNA Names between two different miRBase versions

In *miRBaseConverter* package, there are two ways to conduct the conversion of miRNA Names between two different miRBase versions.

4.1 Solution 1: Global searching and matching

miRBaseConverter package provides the `miRNAVersionConvert()` function to detect all the match miRNA Names with the same Accession in all miRBase historical versions. The conversion result may not match to the unique Name for some miRNAs but it is useful for all possible information retrieval for the miRNAs of interest.

```
library(miRBaseConverter)
data(miRNATest)
miRNANames = miRNATest$miRNA_Name
result1 = miRNAVersionConvert(miRNANames,targetVersion = "v13",exact = TRUE)
## ********************************************
## The multiple matched miRNAs are list below:
##     original           Version v13              Accession
## 1 hsa-let-7c hsa-let-7c&hsa-let-7c MI0000064&MIMAT0000064
## 2  cel-lsy-6   cel-lsy-6&cel-lsy-6 MI0000801&MIMAT0000749
```

```
result1[c(341:345),]
##      OriginalName   TargetName    Accession
## 341  hsa-miR-520b hsa-miR-520b MIMAT0002843
## 342   mtr-miR399c  mtr-miR399c MIMAT0001650
## 343 cel-miR-42-3p   cel-miR-42 MIMAT0000013
## 344   osa-miR529a  osa-miR529a MIMAT0002885
## 345   mtr-miR169j         <NA>         <NA>

result2 = miRNAVersionConvert(miRNANames,targetVersion = "v20",exact = TRUE)
## ********************************************
##
## The multiple matched miRNAs are list below:
##    original         Version v20             Accession
## 1 cel-lsy-6 cel-lsy-6&cel-lsy-6 MI0000801&MIMAT0000749

result2[c(341:345),]
##      OriginalName    TargetName    Accession
## 341  hsa-miR-520b  hsa-miR-520b MIMAT0002843
## 342   mtr-miR399c   mtr-miR399c MIMAT0001650
## 343 cel-miR-42-3p cel-miR-42-3p MIMAT0000013
## 344   osa-miR529a   osa-miR529a MIMAT0002885
## 345   mtr-miR169j   mtr-miR169j MIMAT0013321
```

4.2 Solution 2: miRNA Names conversion with three steps

The miRBase Accession can be a bridge to exactly match miRNA Name between two different miRBase versions. For a group of miRNA Names, users could apply the function `checkMiRNAVersion()` to check the possible miRNA version firstly. Then the miRNA Names accompanying with the version information are mapped to the Accessions using the function `miRNA_NameToAccession()`. In the last step, the Accessions can be easily mapped to the miRNA Names in the target version. This approach can output more exact result than the global searching and matching. [`checkMiRNAVersion()`-> `miRNA_NameToAccession()` -> `miRNA_AccessionToName()`]

```
library(miRBaseConverter)
data(miRNATest)
miRNANames = miRNATest$miRNA_Name

#### Step 1. Check the possible version for miRNAs
data(miRNATest)
miRNANames = miRNATest$miRNA_Name
version=checkMiRNAVersion(miRNANames, verbose = FALSE)

#### Step 2. miRNA Names to miRBase Accessions with the specific version information
result1 = miRNA_NameToAccession(miRNANames,version = version)

#### Step 3. miRBase Accessions to miRNA Names of the target version
result2 = miRNA_AccessionToName(result1[,2],targetVersion = "v22")
result2[c(341:345),]
```

```
##        Accession      TargetName
## 341 MIMAT0002843 hsa-miR-520b-3p
## 342 MIMAT0001650      mtr-miR399c
## 343 MIMAT0000013    cel-miR-42-3p
## 344 MIMAT0002885      osa-miR529a
## 345 MIMAT0011111      mtr-miR169b
```

5 The conversion between Precursor and Mature miRNA

A Precursor miRNA is about 70mer RNA with a stem-loop to form as a hairpin structure. The 5' UTR and/or 3' UTR of the stem-loop can be cleaved by dicer to generate one or two mature miRNAs (about 22 nucleotides). In *miRBaseConverter* package, we provide the functions for conversion between precursors and mature miRNAs.

5.1 Mature miRNA to Precursor

```
library(miRBaseConverter)
data(miRNATest)
miRNANames=miRNATest$miRNA_Name
result1=miRNA_MatureToPrecursor(miRNANames)
## The input miRNA version information: miRBase v18
head(result1)
##      OriginalName      Precursor
## 1   cel-miR-46-3p     cel-mir-46
## 2   cel-miR-81-3p     cel-mir-81
## 3    cel-miR-1817   cel-mir-1817
## 4 hsa-miR-196a-5p hsa-mir-196a-1
## 5  mmu-miR-149-5p    mmu-mir-149
## 6     mtr-miR166d    mtr-MIR166d
```

5.2 Precursor to Mature miRNA

```
library(miRBaseConverter)
miRNANames=c("pma-mir-100a","sko-mir-92a","hsa-mir-6131","mtr-MIR2655i",
"mmu-mir-153","mtr-MIR2592am","mml-mir-1239","xtr-mir-128-2","oan-mir-100",
"mmu-mir-378b","hsa-miR-508-5p","mmu-miR-434-3p")
result2=miRNA_PrecursorToMature(miRNANames)
## The input miRNA version information: miRBase v22
head(result2)
##    OriginalName         Mature1         Mature2
## 1  pma-mir-100a pma-miR-100a-5p pma-miR-100a-3p
## 2   sko-mir-92a     sko-miR-92a            <NA>
## 3  hsa-mir-6131    hsa-miR-6131            <NA>
```

```
## 4  mtr-MIR2655i    mtr-miR2655i            <NA>
## 5   mmu-mir-153  mmu-miR-153-5p  mmu-miR-153-3p
## 6 mtr-MIR2592am   mtr-miR2592am            <NA>
```

6 Retrieve the Family category of miRNAs

miRNAs are manually classified by the single-linkage method to cluster the precursor sequences based on BLAST hits and adjusted manually the clustered families by multiple sequence alignment in miRBase. The family classification is based on a common ancestor for each family representing sequences. Normally, the miRNAs from the same family may possess similar physiological functions in cell metabolism. In *miRBaseConverter* package, the function `checkMiRNAFamily()` is specifically designed for the retrieval information of miRNA families. It can be applied to retrieve the miRNA families and family accessions for a list of miRNAs.

```
library(miRBaseConverter)
## The input is miRNA Accessions
Accessions=miRNATest$Accession
Family_Info1=checkMiRNAFamily(Accessions)
head(Family_Info1)
##      Accession   miRNAName_v21 FamilyAccession  Family
## 1 MIMAT0000017   cel-miR-46-3p     MIPF0000087  mir-46
## 2 MIMAT0000054   cel-miR-81-3p     MIPF0000154  mir-81
## 3 MIMAT0006584    cel-miR-1817            <NA>    <NA>
## 4 MIMAT0000226 hsa-miR-196a-5p     MIPF0000031 mir-196
## 5 MIMAT0000159  mmu-miR-149-5p     MIPF0000274 mir-149
## 6 MIMAT0011068     mtr-miR166d     MIPF0000004  MIR166

##The input is miRNA names
miRNANames=miRNATest$miRNA_Name
version=checkMiRNAVersion(miRNANames,verbose = FALSE)
result=miRNA_NameToAccession(miRNANames,version=version)
Accessions=result$Accession
Family_Info2=checkMiRNAFamily(Accessions)
head(Family_Info2)
##      Accession   miRNAName_v21 FamilyAccession  Family
## 1 MIMAT0000017   cel-miR-46-3p     MIPF0000087  mir-46
## 2 MIMAT0000054   cel-miR-81-3p     MIPF0000154  mir-81
## 3 MIMAT0006584    cel-miR-1817            <NA>    <NA>
## 4 MIMAT0000226 hsa-miR-196a-5p     MIPF0000031 mir-196
## 5 MIMAT0000159  mmu-miR-149-5p     MIPF0000274 mir-149
## 6 MIMAT0011068     mtr-miR166d     MIPF0000004  MIR166
```

7 Retrieve some of detailed miRNA information in miRBase

7.1 Retrieve the Sequence of miRNAs

The miRNA sequence is great important for base alignment in the research of gene regulation. In *miRBaseConverter* package, we provide an efficient tool to batch retrieve the sequence of miRNAs based on the Accessions. It will be great help of automated analyses of sequence alignment between miRNAs and their target molecules.

```
library(miRBaseConverter)
data(miRNATest)
Accessions = miRNATest$Accession
result1 = getMiRNASequence(Accessions,targetVersion = "v13")
head(result1)
##      Accession       miRNASequence_v13
## 1 MIMAT0000017  UGUCAUGGAGUCGCUCUCUUCA
## 2 MIMAT0000054  UGAGAUCAUCGUGAAAGCUAGU
## 3 MIMAT0006584 UAGCCAAUGUCUUCUCUAUCAUG
## 4 MIMAT0000226  UAGGUAGUUUCAUGUUGUUGGG
## 5 MIMAT0000159 UCUGGCUCCGUGUCUUCACUCCC
## 6 MIMAT0011068                    <NA>

result2 = getMiRNASequence(Accessions,targetVersion = "v22")
head(result2)
##      Accession       miRNASequence_v22
## 1 MIMAT0000017  UGUCAUGGAGUCGCUCUCUUCA
## 2 MIMAT0000054  UGAGAUCAUCGUGAAAGCUAGU
## 3 MIMAT0006584 UAGCCAAUGUCUUCUCUAUCAUG
## 4 MIMAT0000226  UAGGUAGUUUCAUGUUGUUGGG
## 5 MIMAT0000159 UCUGGCUCCGUGUCUUCACUCCC
## 6 MIMAT0011068   UCGGGCCAGGCUUCAUCCCCC
```

7.2 Retrieve all the miRBase version information

Currently, the latest miRBase version is 22 which was released in March 2018. In *miRBaseConverter* package, we implement a query function to check all the miRBase version information from miRBase version 1 to version 22. The return includes the information of the defined version names, release dates, the number of defined miRNAs (Entries including Precursors and Mature miRNAs) and the available status in *miRBaseConverter* package.

```
library(miRBaseConverter)
getAllVersionInfo()
##    Version    Date      Status hairpin.precursors matures species
## 1       v1 12/2002 Unavailable                218      NA      NA
## 2     v1_1 01/2003 Unavailable                262      NA      NA
## 3     v1_2 04/2003 Unavailable                295      NA      NA
## 4     v1_3 05/2003 Unavailable                332      NA      NA
```

```
## 5      v1_4 07/2003 Unavailable             345      NA      NA
## 6        v2 07/2003 Unavailable             506      NA      NA
## 7      v2_1 09/2003 Unavailable             558      NA      NA
## 8      v2_2 11/2003 Unavailable             593      NA      NA
## 9        v3 01/2004 Unavailable             719      NA      NA
## 10     v3_1 04/2004 Unavailable             899      NA      NA
## 11       v4 07/2004 Unavailable            1185      NA      NA
## 12       v5 09/2004 Unavailable            1345      NA      NA
## 13     v5_1 12/2004 Unavailable            1420      NA      NA
## 14       v6 04/2005   Available            1650    1591      21
## 15       v7 06/2005 Unavailable            2909      NA      NA
## 16     v7_1 10/2005   Available            3424    3102      40
## 17       v8 02/2006   Available            3518    3229      41
## 18     v8_1 05/2006   Available            3963    3685      44
## 19     v8_2 07/2006   Available            4039    3834      45
## 20       v9 10/2006   Available            4361    4167      49
## 21     v9_1 02/2007   Available            4449    4274      49
## 22     v9_2 05/2007   Available            4584    4430      55
## 23      v10 08/2007   Available            5071    4922      58
## 24    v10_1 12/2007   Available            5395    5234      66
## 25      v11 04/2008   Available            6396    6211      72
## 26      v12 09/2008   Available            8619    8273      87
## 27      v13 03/2009   Available            9539    9169     103
## 28      v14 09/2009   Available           10883   10581     115
## 29      v15 04/2010   Available           14197   15632     133
## 30      v16 08/2010   Available           15172   17341     142
## 31      v17 04/2011   Available           16772   19724     153
## 32      v18 11/2011   Available           18226   21643     168
## 33      v19 08/2012   Available           21264   25141     193
## 34      v20 06/2013   Available           24521   30424     206
## 35      v21 06/2014   Available           28645   35828     223
## 36      v22 03/2018   Available           38589   48885     271
```

7.3 Retrieve all the available species in miRBase

miRBase has defined the miRNAs for hundreds of species. In *miRBaseConverter* package, users can apply the function `getAllSpecies()` to check the abbreviation and the full name of the available species.

```
library(miRBaseConverter)
allSpecies=getAllSpecies()
head(allSpecies)
##   Species division                          name
## 1     aqu      AQU      Amphimedon queenslandica
## 2     nve      NVE        Nematostella vectensis
## 3     hma      HMA          Hydra magnipapillata
## 4     sko      SKO      Saccoglossus kowalevskii
## 5     spu      SPU Strongylocentrotus purpuratus
## 6     cin      CIN            Ciona intestinalis
```

```
##                                                    tree NCBI-taxid
## 1                                      Metazoa;Porifera;     400682
## 2                                      Metazoa;Cnidaria;      45351
## 3                                      Metazoa;Cnidaria;       6085
## 4          Metazoa;Bilateria;Deuterostoma;Hemichordata;      10224
## 5         Metazoa;Bilateria;Deuterostoma;Echinodermata;       7668
## 6 Metazoa;Bilateria;Deuterostoma;Chordata;Urochordata;       7719
```

7.4 Retrieve all the available miRNAs in the specified miRBase version

In *miRBaseConverter* package, the function `getAllMiRNAs()` can be applied to get all miRNAs which are defined in each available miRBase version. Meanwhile, users can use the control parameters to custom the species and miRNA type in the output.

```
library(miRBaseConverter)
miRNAs=getAllMiRNAs(version="v22", type="all", species="hsa")
head(miRNAs)
##      Accession            Name
## 1    MI0000060    hsa-let-7a-1
## 2    MI0000061    hsa-let-7a-2
## 3 MIMAT0010195 hsa-let-7a-2-3p
## 4    MI0000062    hsa-let-7a-3
## 5 MIMAT0004481   hsa-let-7a-3p
## 6 MIMAT0000062   hsa-let-7a-5p
##                                                                             Sequence
## 1 UGGGAUGAGGUAGUAGGUUGUAUAGUUUUAGGGUCACACCCACCACUGGGAGAUAACUAUACAAUCUACUGUCUUUCCUA
## 2         AGGUUGAGGUAGUAGGUUGUAUAGUUUAGAAUUACAUCAAGGGAGAUAACUGUACAGCCUCCUAGCUUUCCU
## 3                                                                  CUGUACAGCCUCCUAGCUUUCC
## 4       GGGUGAGGUAGUAGGUUGUAUAGUUUGGGGCUCUGCCCUGCUAUGGGAUAACUAUACAAUCUACUGUCUUUCCU
## 5                                                                  CUAUACAAUCUACUGUCUUUC
## 6                                                                  UGAGGUAGUAGGUUGUAUAGUU
```

7.5 Retrieve all the history information of a single miRNA

In some applications, users may want to have a comprehensive investigation of a miRNA about the Name, Accession, Sequence, Precursor and mature miRNA information. The *miRBaseConverter* package embeds a useful function `getMiRNAHistory()` to retrieve all the detailed miRNA information in all miRBase historic version. Users can get a comprehensive overview of the miRNA.

```
#### 1. The input is a miRNA Name
name = "hsa-miR-26b-5p"
result1 = miRNA_NameToAccession(name)
Accession = result1$Accession
result2 = getMiRNAHistory(Accession)
result2
##         Precursor
```

```
## v6    hsa-mir-26b
## v7_1  hsa-mir-26b
## v8    hsa-mir-26b
## v8_1  hsa-mir-26b
## v8_2  hsa-mir-26b
## v9    hsa-mir-26b
## v9_1  hsa-mir-26b
## v9_2  hsa-mir-26b
## v10   hsa-mir-26b
## v10_1 hsa-mir-26b
## v11   hsa-mir-26b
## v12   hsa-mir-26b
## v13   hsa-mir-26b
## v14   hsa-mir-26b
## v15   hsa-mir-26b
## v16   hsa-mir-26b
## v17   hsa-mir-26b
## v18   hsa-mir-26b
## v19   hsa-mir-26b
## v20   hsa-mir-26b
## v21   hsa-mir-26b
## v22   hsa-mir-26b
##                                                                       PrecursorSequence
## v6    CCGGGACCCAGUUCAAGUAAUUCAGGAUAGGUUGUGUGCUGUCCAGCCUGUUCUCCAUUACUUGGCUCGGGGACCGG
## v7_1  CCGGGACCCAGUUCAAGUAAUUCAGGAUAGGUUGUGUGCUGUCCAGCCUGUUCUCCAUUACUUGGCUCGGGGACCGG
## v8    CCGGGACCCAGUUCAAGUAAUUCAGGAUAGGUUGUGUGCUGUCCAGCCUGUUCUCCAUUACUUGGCUCGGGGACCGG
## v8_1  CCGGGACCCAGUUCAAGUAAUUCAGGAUAGGUUGUGUGCUGUCCAGCCUGUUCUCCAUUACUUGGCUCGGGGACCGG
## v8_2  CCGGGACCCAGUUCAAGUAAUUCAGGAUAGGUUGUGUGCUGUCCAGCCUGUUCUCCAUUACUUGGCUCGGGGACCGG
## v9    CCGGGACCCAGUUCAAGUAAUUCAGGAUAGGUUGUGUGCUGUCCAGCCUGUUCUCCAUUACUUGGCUCGGGGACCGG
## v9_1  CCGGGACCCAGUUCAAGUAAUUCAGGAUAGGUUGUGUGCUGUCCAGCCUGUUCUCCAUUACUUGGCUCGGGGACCGG
## v9_2  CCGGGACCCAGUUCAAGUAAUUCAGGAUAGGUUGUGUGCUGUCCAGCCUGUUCUCCAUUACUUGGCUCGGGGACCGG
## v10   CCGGGACCCAGUUCAAGUAAUUCAGGAUAGGUUGUGUGCUGUCCAGCCUGUUCUCCAUUACUUGGCUCGGGGACCGG
## v10_1 CCGGGACCCAGUUCAAGUAAUUCAGGAUAGGUUGUGUGCUGUCCAGCCUGUUCUCCAUUACUUGGCUCGGGGACCGG
## v11   CCGGGACCCAGUUCAAGUAAUUCAGGAUAGGUUGUGUGCUGUCCAGCCUGUUCUCCAUUACUUGGCUCGGGGACCGG
## v12   CCGGGACCCAGUUCAAGUAAUUCAGGAUAGGUUGUGUGCUGUCCAGCCUGUUCUCCAUUACUUGGCUCGGGGACCGG
## v13   CCGGGACCCAGUUCAAGUAAUUCAGGAUAGGUUGUGUGCUGUCCAGCCUGUUCUCCAUUACUUGGCUCGGGGACCGG
## v14   CCGGGACCCAGUUCAAGUAAUUCAGGAUAGGUUGUGUGCUGUCCAGCCUGUUCUCCAUUACUUGGCUCGGGGACCGG
## v15   CCGGGACCCAGUUCAAGUAAUUCAGGAUAGGUUGUGUGCUGUCCAGCCUGUUCUCCAUUACUUGGCUCGGGGACCGG
## v16   CCGGGACCCAGUUCAAGUAAUUCAGGAUAGGUUGUGUGCUGUCCAGCCUGUUCUCCAUUACUUGGCUCGGGGACCGG
## v17   CCGGGACCCAGUUCAAGUAAUUCAGGAUAGGUUGUGUGCUGUCCAGCCUGUUCUCCAUUACUUGGCUCGGGGACCGG
## v18   CCGGGACCCAGUUCAAGUAAUUCAGGAUAGGUUGUGUGCUGUCCAGCCUGUUCUCCAUUACUUGGCUCGGGGACCGG
## v19   CCGGGACCCAGUUCAAGUAAUUCAGGAUAGGUUGUGUGCUGUCCAGCCUGUUCUCCAUUACUUGGCUCGGGGACCGG
## v20   CCGGGACCCAGUUCAAGUAAUUCAGGAUAGGUUGUGUGCUGUCCAGCCUGUUCUCCAUUACUUGGCUCGGGGACCGG
## v21   CCGGGACCCAGUUCAAGUAAUUCAGGAUAGGUUGUGUGCUGUCCAGCCUGUUCUCCAUUACUUGGCUCGGGGACCGG
## v22   CCGGGACCCAGUUCAAGUAAUUCAGGAUAGGUUGUGUGCUGUCCAGCCUGUUCUCCAUUACUUGGCUCGGGGACCGG
##              Mature1        Mature1Sequence        Mature2
## v6       hsa-miR-26b UUCAAGUAAUUCAGGAUAGGUU           <NA>
## v7_1     hsa-miR-26b UUCAAGUAAUUCAGGAUAGGUU           <NA>
## v8       hsa-miR-26b UUCAAGUAAUUCAGGAUAGGUU           <NA>
## v8_1     hsa-miR-26b UUCAAGUAAUUCAGGAUAGGUU           <NA>
## v8_2     hsa-miR-26b UUCAAGUAAUUCAGGAUAGGUU           <NA>
```

```
## v9       hsa-miR-26b UUCAAGUAAUUCAGGAUAGGUU           <NA>
## v9_1     hsa-miR-26b UUCAAGUAAUUCAGGAUAGGUU           <NA>
## v9_2     hsa-miR-26b UUCAAGUAAUUCAGGAUAGGUU           <NA>
## v10      hsa-miR-26b  UUCAAGUAAUUCAGGAUAGGU   hsa-miR-26b*
## v10_1    hsa-miR-26b  UUCAAGUAAUUCAGGAUAGGU   hsa-miR-26b*
## v11      hsa-miR-26b  UUCAAGUAAUUCAGGAUAGGU   hsa-miR-26b*
## v12      hsa-miR-26b  UUCAAGUAAUUCAGGAUAGGU   hsa-miR-26b*
## v13      hsa-miR-26b  UUCAAGUAAUUCAGGAUAGGU   hsa-miR-26b*
## v14      hsa-miR-26b  UUCAAGUAAUUCAGGAUAGGU   hsa-miR-26b*
## v15      hsa-miR-26b  UUCAAGUAAUUCAGGAUAGGU   hsa-miR-26b*
## v16      hsa-miR-26b  UUCAAGUAAUUCAGGAUAGGU   hsa-miR-26b*
## v17      hsa-miR-26b  UUCAAGUAAUUCAGGAUAGGU   hsa-miR-26b*
## v18   hsa-miR-26b-5p  UUCAAGUAAUUCAGGAUAGGU hsa-miR-26b-3p
## v19   hsa-miR-26b-5p  UUCAAGUAAUUCAGGAUAGGU hsa-miR-26b-3p
## v20   hsa-miR-26b-5p  UUCAAGUAAUUCAGGAUAGGU hsa-miR-26b-3p
## v21   hsa-miR-26b-5p  UUCAAGUAAUUCAGGAUAGGU hsa-miR-26b-3p
## v22   hsa-miR-26b-5p  UUCAAGUAAUUCAGGAUAGGU hsa-miR-26b-3p
##              Mature2Sequence    Status
## v6                      <NA> UNCHANGED
## v7_1                    <NA>      <NA>
## v8                      <NA> UNCHANGED
## v8_1                    <NA> UNCHANGED
## v8_2                    <NA> UNCHANGED
## v9                      <NA> UNCHANGED
## v9_1                    <NA> UNCHANGED
## v9_2                    <NA> UNCHANGED
## v10   CCUGUUCUCCAUUACUUGGCUC UNCHANGED
## v10_1 CCUGUUCUCCAUUACUUGGCUC UNCHANGED
## v11   CCUGUUCUCCAUUACUUGGCUC UNCHANGED
## v12   CCUGUUCUCCAUUACUUGGCUC UNCHANGED
## v13   CCUGUUCUCCAUUACUUGGCUC UNCHANGED
## v14   CCUGUUCUCCAUUACUUGGCUC UNCHANGED
## v15   CCUGUUCUCCAUUACUUGGCUC UNCHANGED
## v16   CCUGUUCUCCAUUACUUGGCUC UNCHANGED
## v17   CCUGUUCUCCAUUACUUGGCUC UNCHANGED
## v18   CCUGUUCUCCAUUACUUGGCUC UNCHANGED
## v19   CCUGUUCUCCAUUACUUGGCUC UNCHANGED
## v20   CCUGUUCUCCAUUACUUGGCUC UNCHANGED
## v21   CCUGUUCUCCAUUACUUGGCUC UNCHANGED
## v22    CCUGUUCUCCAUUACUUGGCU UNCHANGED
```

```
#### 2. The input is miRNA Accession Id
Accession = "MIMAT0000765"
result3 = getMiRNAHistory(Accession)
result3
##         Precursor
## v6    hsa-mir-335
## v7_1  hsa-mir-335
## v8    hsa-mir-335
## v8_1  hsa-mir-335
## v8_2  hsa-mir-335
```

```
## v9    hsa-mir-335
## v9_1  hsa-mir-335
## v9_2  hsa-mir-335
## v10   hsa-mir-335
## v10_1 hsa-mir-335
## v11   hsa-mir-335
## v12   hsa-mir-335
## v13   hsa-mir-335
## v14   hsa-mir-335
## v15   hsa-mir-335
## v16   hsa-mir-335
## v17   hsa-mir-335
## v18   hsa-mir-335
## v19   hsa-mir-335
## v20   hsa-mir-335
## v21   hsa-mir-335
## v22   hsa-mir-335
##                                                                                   PrecursorSequence
## v6    UGUUUUGAGCGGGGGUCAAGAGCAAUAACGAAAAAUGUUUGUCAUAAACCGUUUUUCAUUAUUGCUCCUGACCUCCUCUCAUUUGCUAUAUUCA
## v7_1  UGUUUUGAGCGGGGGUCAAGAGCAAUAACGAAAAAUGUUUGUCAUAAACCGUUUUUCAUUAUUGCUCCUGACCUCCUCUCAUUUGCUAUAUUCA
## v8    UGUUUUGAGCGGGGGUCAAGAGCAAUAACGAAAAAUGUUUGUCAUAAACCGUUUUUCAUUAUUGCUCCUGACCUCCUCUCAUUUGCUAUAUUCA
## v8_1  UGUUUUGAGCGGGGGUCAAGAGCAAUAACGAAAAAUGUUUGUCAUAAACCGUUUUUCAUUAUUGCUCCUGACCUCCUCUCAUUUGCUAUAUUCA
## v8_2  UGUUUUGAGCGGGGGUCAAGAGCAAUAACGAAAAAUGUUUGUCAUAAACCGUUUUUCAUUAUUGCUCCUGACCUCCUCUCAUUUGCUAUAUUCA
## v9    UGUUUUGAGCGGGGGUCAAGAGCAAUAACGAAAAAUGUUUGUCAUAAACCGUUUUUCAUUAUUGCUCCUGACCUCCUCUCAUUUGCUAUAUUCA
## v9_1  UGUUUUGAGCGGGGGUCAAGAGCAAUAACGAAAAAUGUUUGUCAUAAACCGUUUUUCAUUAUUGCUCCUGACCUCCUCUCAUUUGCUAUAUUCA
## v9_2  UGUUUUGAGCGGGGGUCAAGAGCAAUAACGAAAAAUGUUUGUCAUAAACCGUUUUUCAUUAUUGCUCCUGACCUCCUCUCAUUUGCUAUAUUCA
## v10   UGUUUUGAGCGGGGGUCAAGAGCAAUAACGAAAAAUGUUUGUCAUAAACCGUUUUUCAUUAUUGCUCCUGACCUCCUCUCAUUUGCUAUAUUCA
## v10_1 UGUUUUGAGCGGGGGUCAAGAGCAAUAACGAAAAAUGUUUGUCAUAAACCGUUUUUCAUUAUUGCUCCUGACCUCCUCUCAUUUGCUAUAUUCA
## v11   UGUUUUGAGCGGGGGUCAAGAGCAAUAACGAAAAAUGUUUGUCAUAAACCGUUUUUCAUUAUUGCUCCUGACCUCCUCUCAUUUGCUAUAUUCA
## v12   UGUUUUGAGCGGGGGUCAAGAGCAAUAACGAAAAAUGUUUGUCAUAAACCGUUUUUCAUUAUUGCUCCUGACCUCCUCUCAUUUGCUAUAUUCA
## v13   UGUUUUGAGCGGGGGUCAAGAGCAAUAACGAAAAAUGUUUGUCAUAAACCGUUUUUCAUUAUUGCUCCUGACCUCCUCUCAUUUGCUAUAUUCA
## v14   UGUUUUGAGCGGGGGUCAAGAGCAAUAACGAAAAAUGUUUGUCAUAAACCGUUUUUCAUUAUUGCUCCUGACCUCCUCUCAUUUGCUAUAUUCA
## v15   UGUUUUGAGCGGGGGUCAAGAGCAAUAACGAAAAAUGUUUGUCAUAAACCGUUUUUCAUUAUUGCUCCUGACCUCCUCUCAUUUGCUAUAUUCA
## v16   UGUUUUGAGCGGGGGUCAAGAGCAAUAACGAAAAAUGUUUGUCAUAAACCGUUUUUCAUUAUUGCUCCUGACCUCCUCUCAUUUGCUAUAUUCA
## v17   UGUUUUGAGCGGGGGUCAAGAGCAAUAACGAAAAAUGUUUGUCAUAAACCGUUUUUCAUUAUUGCUCCUGACCUCCUCUCAUUUGCUAUAUUCA
## v18   UGUUUUGAGCGGGGGUCAAGAGCAAUAACGAAAAAUGUUUGUCAUAAACCGUUUUUCAUUAUUGCUCCUGACCUCCUCUCAUUUGCUAUAUUCA
## v19   UGUUUUGAGCGGGGGUCAAGAGCAAUAACGAAAAAUGUUUGUCAUAAACCGUUUUUCAUUAUUGCUCCUGACCUCCUCUCAUUUGCUAUAUUCA
## v20   UGUUUUGAGCGGGGGUCAAGAGCAAUAACGAAAAAUGUUUGUCAUAAACCGUUUUUCAUUAUUGCUCCUGACCUCCUCUCAUUUGCUAUAUUCA
## v21   UGUUUUGAGCGGGGGUCAAGAGCAAUAACGAAAAAUGUUUGUCAUAAACCGUUUUUCAUUAUUGCUCCUGACCUCCUCUCAUUUGCUAUAUUCA
## v22   UGUUUUGAGCGGGGGUCAAGAGCAAUAACGAAAAAUGUUUGUCAUAAACCGUUUUUCAUUAUUGCUCCUGACCUCCUCUCAUUUGCUAUAUUCA
##              Mature1         Mature1Sequence        Mature2
## v6       hsa-miR-335 UCAAGAGCAAUAACGAAAAAUGU           <NA>
## v7_1     hsa-miR-335 UCAAGAGCAAUAACGAAAAAUGU           <NA>
## v8       hsa-miR-335 UCAAGAGCAAUAACGAAAAAUGU           <NA>
## v8_1     hsa-miR-335 UCAAGAGCAAUAACGAAAAAUGU           <NA>
## v8_2     hsa-miR-335 UCAAGAGCAAUAACGAAAAAUGU           <NA>
## v9       hsa-miR-335 UCAAGAGCAAUAACGAAAAAUGU           <NA>
## v9_1     hsa-miR-335 UCAAGAGCAAUAACGAAAAAUGU           <NA>
## v9_2     hsa-miR-335 UCAAGAGCAAUAACGAAAAAUGU           <NA>
## v10      hsa-miR-335 UCAAGAGCAAUAACGAAAAAUGU   hsa-miR-335*
## v10_1    hsa-miR-335 UCAAGAGCAAUAACGAAAAAUGU   hsa-miR-335*
```

```
## v11      hsa-miR-335 UCAAGAGCAAUAACGAAAAAUGU   hsa-miR-335*
## v12      hsa-miR-335 UCAAGAGCAAUAACGAAAAAUGU   hsa-miR-335*
## v13      hsa-miR-335 UCAAGAGCAAUAACGAAAAAUGU   hsa-miR-335*
## v14      hsa-miR-335 UCAAGAGCAAUAACGAAAAAUGU   hsa-miR-335*
## v15      hsa-miR-335 UCAAGAGCAAUAACGAAAAAUGU   hsa-miR-335*
## v16      hsa-miR-335 UCAAGAGCAAUAACGAAAAAUGU   hsa-miR-335*
## v17      hsa-miR-335 UCAAGAGCAAUAACGAAAAAUGU   hsa-miR-335*
## v18   hsa-miR-335-5p UCAAGAGCAAUAACGAAAAAUGU hsa-miR-335-3p
## v19   hsa-miR-335-5p UCAAGAGCAAUAACGAAAAAUGU hsa-miR-335-3p
## v20   hsa-miR-335-5p UCAAGAGCAAUAACGAAAAAUGU hsa-miR-335-3p
## v21   hsa-miR-335-5p UCAAGAGCAAUAACGAAAAAUGU hsa-miR-335-3p
## v22   hsa-miR-335-5p UCAAGAGCAAUAACGAAAAAUGU hsa-miR-335-3p
##             Mature2Sequence    Status
## v6                     <NA> UNCHANGED
## v7_1                   <NA>      <NA>
## v8                     <NA> UNCHANGED
## v8_1                   <NA> UNCHANGED
## v8_2                   <NA> UNCHANGED
## v9                     <NA> UNCHANGED
## v9_1                   <NA> UNCHANGED
## v9_2                   <NA> UNCHANGED
## v10   UUUUUCAUUAUUGCUCCUGACC UNCHANGED
## v10_1 UUUUUCAUUAUUGCUCCUGACC UNCHANGED
## v11   UUUUUCAUUAUUGCUCCUGACC UNCHANGED
## v12   UUUUUCAUUAUUGCUCCUGACC UNCHANGED
## v13   UUUUUCAUUAUUGCUCCUGACC UNCHANGED
## v14   UUUUUCAUUAUUGCUCCUGACC UNCHANGED
## v15   UUUUUCAUUAUUGCUCCUGACC UNCHANGED
## v16   UUUUUCAUUAUUGCUCCUGACC UNCHANGED
## v17   UUUUUCAUUAUUGCUCCUGACC UNCHANGED
## v18   UUUUUCAUUAUUGCUCCUGACC UNCHANGED
## v19   UUUUUCAUUAUUGCUCCUGACC UNCHANGED
## v20   UUUUUCAUUAUUGCUCCUGACC UNCHANGED
## v21   UUUUUCAUUAUUGCUCCUGACC UNCHANGED
## v22   UUUUUCAUUAUUGCUCCUGACC UNCHANGED
```

7.6 Retrieve the data table for the specified miRBase version

There is a data table for presenting the detailed information of the defined miRNAs in each miRBase version. In *miRBaseConverter* package, we provide a function `getMiRNATable()` to return the miRNA data table for each miRBase version. Meanwhile, users can use the control parameter to custom the species in output.

```
library(miRBaseConverter)
miRNA_Tab=getMiRNATable(version="v22",species="hsa")
head(miRNA_Tab)
##    Precursor_Acc    Precursor    Status
## 58     MI0000060 hsa-let-7a-1 UNCHANGED
## 59     MI0000061 hsa-let-7a-2 UNCHANGED
```

```
## 60     MI0000062 hsa-let-7a-3 UNCHANGED
## 61     MI0000063   hsa-let-7b UNCHANGED
## 62     MI0000064   hsa-let-7c UNCHANGED
## 63     MI0000065   hsa-let-7d UNCHANGED
##                                                                                       Precursor_Seq
## 58        UGGGAUGAGGUAGUAGGUUGUAUAGUUUUAGGGUCACACCCACCACUGGGAGAUAACUAUACAAUCUACUGUCUUUCCUA
## 59                AGGUUGAGGUAGUAGGUUGUAUAGUUUAGAAUUACAUCAAGGGAGAUAACUGUACAGCCUCCUAGCUUUCCU
## 60              GGGUGAGGUAGUAGGUUGUAUAGUUUGGGGCUCUGCCCUGCUAUGGGAUAACUAUACAAUCUACUGUCUUUCCU
## 61     CGGGGUGAGGUAGUAGGUUGUGUGGUUUCAGGGCAGUGAUGUUGCCCCUCGGAAGAUAACUAUACAACCUACUGCCUUCCCUG
## 62    GCAUCCGGGUUGAGGUAGUAGGUUGUAUGGUUUAGAGUUACACCCUGGGAGUUAACUGUACAACCUUCUAGCUUUCCUUGGAGC
## 63 CCUAGGAAGAGGUAGUAGGUUGCAUAGUUUUAGGGCAGGGAUUUUGCCCACAAGGAGGUAACUAUACGACCUGCUGCCUUUCUUAGG
##     Mature1_Acc       Mature1            Mature1_Seq  Mature2_Acc
## 58 MIMAT0000062 hsa-let-7a-5p UGAGGUAGUAGGUUGUAUAGUU MIMAT0004481
## 59 MIMAT0000062 hsa-let-7a-5p UGAGGUAGUAGGUUGUAUAGUU MIMAT0010195
## 60 MIMAT0000062 hsa-let-7a-5p UGAGGUAGUAGGUUGUAUAGUU MIMAT0004481
## 61 MIMAT0000063 hsa-let-7b-5p UGAGGUAGUAGGUUGUGUGGUU MIMAT0004482
## 62 MIMAT0000064 hsa-let-7c-5p UGAGGUAGUAGGUUGUAUGGUU MIMAT0026472
## 63 MIMAT0000065 hsa-let-7d-5p AGAGGUAGUAGGUUGCAUAGUU MIMAT0004484
##            Mature2            Mature2_Seq
## 58   hsa-let-7a-3p  CUAUACAAUCUACUGUCUUUC
## 59 hsa-let-7a-2-3p CUGUACAGCCUCCUAGCUUUCC
## 60   hsa-let-7a-3p  CUAUACAAUCUACUGUCUUUC
## 61   hsa-let-7b-3p CUAUACAACCUACUGCCUUCCC
## 62   hsa-let-7c-3p CUGUACAACCUUCUAGCUUUCC
## 63   hsa-let-7d-3p CUAUACGACCUGCUGCCUUUCU
```

8 The online retrieving of miRNA information

8.1 Open the miRNA webpages in miRBase

In *miRBaseConverter* package, the function `goTo_miRBase()` redirects users to the miRBase webpages of some specified miRNAs.

```
library(miRBaseConverter)
Accessions=miRNATest$Accession[1:10]
goTo_miRBase(Accessions)
```

8.2 Open the miRNA family webpages in miRBase

In *miRBaseConverter* package, the function `goTo_miRNAFamily()` redirects users to the miRBase miRNA family webpages of some specified miRNA families.

```
library(miRBaseConverter)
Accessions=miRNATest$Accession
Family_Info=checkMiRNAFamily(Accessions)
FamilyAccessions=Family_Info$FamilyAccession[1:15]
goTo_miRNAFamily(FamilyAccessions)
```

9 Conclusion

The R/Bioconductor package ***miRBaseConverter*** provides a suite of tools for checking miRNA Name, Accession, Sequence, Species, Version, Hisotry and Family information in different miRBase versions. We wish that the ***miRBaseConverter*** package could be a useful tool for miRNA research community and help to speed up the studies of miRNAs.
